# A Fast Contour Detection Model Inspired by Biological Mechanisms in Primary Vision System

**DOI:** 10.3389/fncom.2018.00028

**Published:** 2018-04-30

**Authors:** Xiaomei Kang, Qingqun Kong, Yi Zeng, Bo Xu

**Affiliations:** ^1^Research Center for Brain-Inspired Intelligence, Institute of Automation, Chinese Academy of Sciences, Beijing, China; ^2^University of Chinese Academy of Sciences, Beijing, China; ^3^Center for Excellence in Brain Science and Intelligence Technology, Chinese Academy of Science, Shanghai, China; ^4^National Laboratory of Pattern Recognition, Institute of Automation, Chinese Academy of Sciences, Beijing, China

**Keywords:** primary visual system, biological mechanism, contour detection, prior filtering, uniform sampling, sparse coding

## Abstract

Compared to computer vision systems, the human visual system is more fast and accurate. It is well accepted that V1 neurons can well encode contour information. There are plenty of computational models about contour detection based on the mechanism of the V1 neurons. Multiple-cue inhibition operator is one well-known model, which is based on the mechanism of V1 neurons' non-classical receptive fields. However, this model is time-consuming and noisy. To solve these two problems, we propose an improved model which integrates some additional other mechanisms of the primary vision system. Firstly, based on the knowledge that the salient contours only occupy a small portion of the whole image, the prior filtering is introduced to decrease the running time. Secondly, based on the physiological finding that nearby neurons often have highly correlated responses and thus include redundant information, we adopt the uniform samplings to speed up the algorithm. Thirdly, sparse coding is introduced to suppress the unwanted noises. Finally, to validate the performance, we test it on Berkeley Segmentation Data Set. The results show that the improved model can decrease running time as well as keep the accuracy of the contour detection.

## Introduction

Contour detection is a fundamental and critical step in computer vision tasks. Recent years, several models have been proposed to detect the contours, such as local differential (Canny, 1986), statistical methods (Konishi et al., 2003), relaxation labeling (Rosenfeld et al., 1976), active contours (Caselles et al., 1997). These methods achieved good performance in some scenes. However, they cannot extract salient contours from complex scenes as intelligent as the human.

Hubel and Wiesel (1959) revealed that the majority of V1 cells have high orientation selectivity. The result showed that cells did not respond to light stimuli which covered the majority of the animal's visual fields, whereas responded most strongly to the light spot stimuli with one specific orientation. The specific orientation is the preferred orientation for the neuron. This mechanism is very suitable for detecting edges produced by the light and dark contrast.

In the primary visual cortex, a region around the classical receptive field (CRF) of one neuron was called the non-classical receptive field (non-CRF) (Allman et al., 1985). The non-CRF played a modulatory effect on signals within the CRF, which was called center-surround interaction (Fitzpatrick, 2000; Jones et al., 2001). The strength of the negative correlation decreased with the differences between the features within the center and that within the surround (Shen et al., 2007). The inhibition intensity was minimal when features within the CRF and non-CRF were completely different.

Based on the biological mechanisms mentioned above, some models have been proposed. Most were based on the center-surround mechanism, and focused on the single feature for edge suppression (Li, 1998; Grigorescu et al., 2003; Petkov and Westenberg, 2003; Ursino and La Cara, 2004; Papari et al., 2007; Tang et al., 2007a,b; La Cara and Ursino, 2008; Long and Li, 2008; Zeng et al., 2011; Yang et al., 2013). And some models integrated multiple features such as Pb (Martin et al., 2004) algorithm, gPb (Maire et al., 2008), and mPb (Ren, 2008). All these methods needed a supervised learning phase to obtain a good performance.

MCI model (Multiple-cue inhibition operator) (Yang et al., 2014) was proposed based on the above-mentioned biological mechanisms, which integrated multiple features using a multi-scale strategy without adopting supervised learning. Compared with other models, this model showed a competitive performance. However, the biologically inspired method was time-consuming and noisy, due to its computational mechanisms of inhibitory responses.

In this paper, we propose a fast contour extraction model based on MCI, which is named speed MCI (sMCI). The prior filtering and uniform sampling are introduced to accelerate the computation of inhibitory responses. Based on biological or behavioral mechanisms, we obtain the whole inhibitory responses with weights of partial pixels to improve the computational efficiency. Besides, the sparseness is computed to exclude redundant information.

The remaining of this paper is organized as follows. Section Methods presents original MCI and the improved model. In section Experiments and Results, the performance of the improved model is validated on BSDS500 dataset and compared with MCI. Discussion and conclusion are given in section Discussion.

## Methods

In this section, we first briefly review MCI and analyze its problems based on the experimental results. Then, we propose an improved model, sMCI, to solve the problem of MCI.

### The MCI model

**Figure 1:**
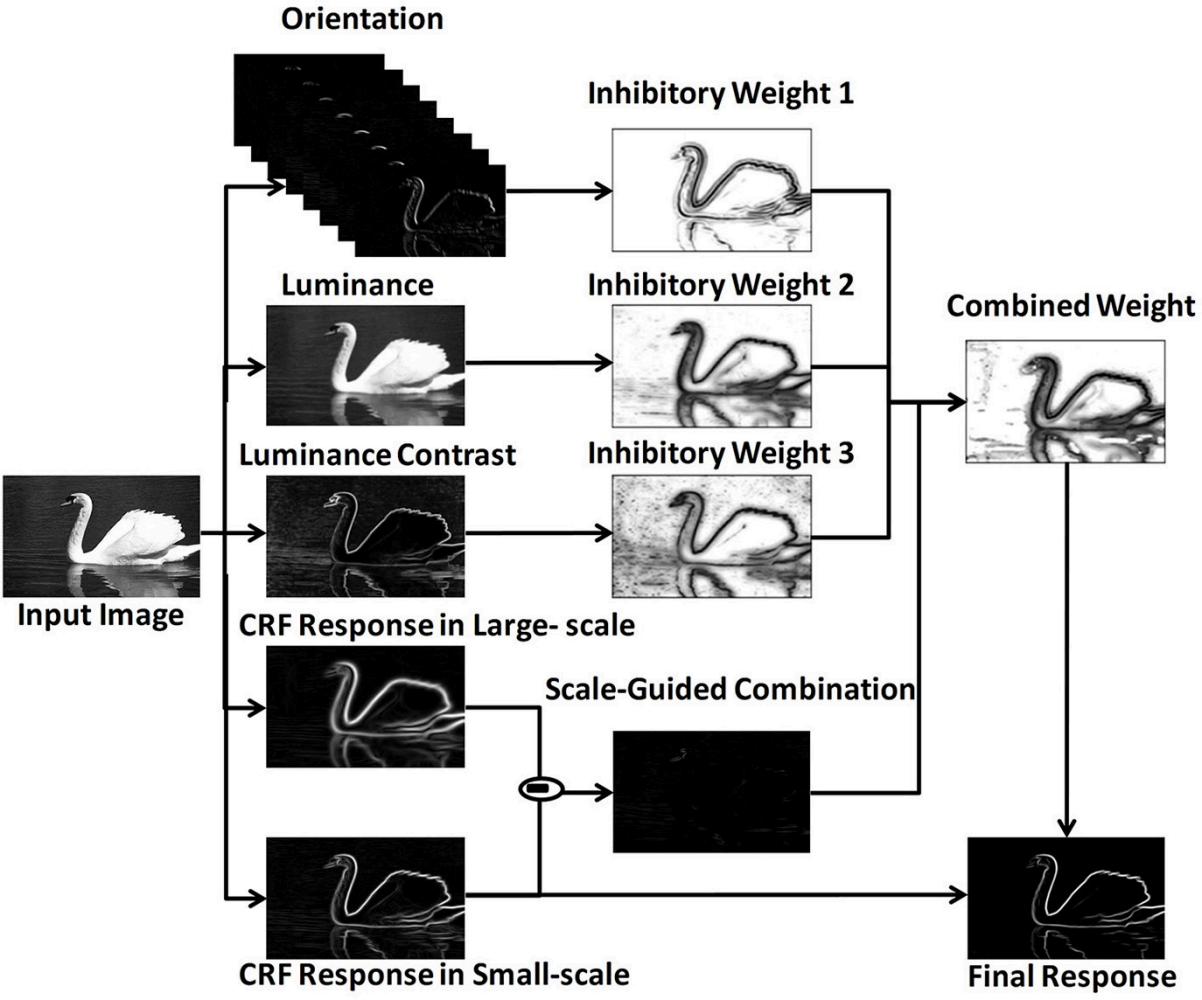
MCI framework revised from Yang et al. (2014).

The MCI algorithm (Yang et al., 2014) was proposed to extract salient contours with the center-surround mechanism. To combine multiple features, the model adopted a scale-guided combination strategy. The framework was shown in Figure 1.

Firstly, the response of one orientation-selective V1 neuron in CRF was calculated. For an input image I(x, y), the response e_i_(x, y; θ_i_, σ) was represented by the derivative of 2D Gaussian function correlated with preferred orientation θ_i_ and scale σ. After a winner - take - all strategy over N_θ_ different preferred orientations, the final CRF response E(x, y; σ) was calculated as in Equation (2),

(1) $$\;{\text{e}}_{\text{i}}\left(\text{x},\;\text{y};\theta_{\text{i}},\;\sigma \right)=\left|\text{I}\left(\text{x},\text{y}\right)*\frac{\partial \text{g}(\tilde{\text{x}},\tilde{\text{y}};\theta_{\text{i}},\sigma )}{\partial \tilde{\text{x}}}\right|$$

(2) $$\text{E}\left(\text{x},\text{y};\sigma \right)=\max\left\{{\text{e}}_{\text{i}}\left(\text{x},\text{y};\theta_{\text{i}},\sigma \right)|\text{i}=1,2,\;\ldots ,{\text{N}}_{\theta }\right\}$$

Secondly, the local features were extracted, including orientation Θ (x, y), luminance L(x, y)and luminance contrast C(x, y). The computational equations of these features were shown in Equations (3) - (5), in which ω(x_i_,y_i_) was a raised cosine weighted window, S_xy_ represented the local square window, and $\mu =\;\underset{\left({\text{x}}_{\text{i}},y_{i}\right)\in {\text{S}}_{\text{xy}}}{\sum }\omega \left({\text{x}}_{\text{i}},y_{i}\right)$.

(3) $$\Theta \left(\text{x},\text{y}\right)={\left[{\text{e}}_{1},{\text{e}}_{2},\ldots ,e_{{\text{N}}_{\theta }}\right]}_{\left(\text{x},\text{y}\right)}^{\text{T}}$$

(4) $$\text{L}\left(\text{x},\text{y}\right)=\frac{1}{\mu }\sum_{\left({\text{x}}_{\text{i}},{\text{y}}_{\text{i}}\right)\in {\text{S}}_{\text{xy}}}\omega \left({\text{x}}_{\text{i}},{\text{y}}_{\text{i}}\right)\text{I}\left({\text{x}}_{\text{i}},{\text{y}}_{\text{i}}\right)$$

(5) $$\text{C}\left(\text{x},\text{y}\right)=\sqrt{\frac{1}{\mu }\sum_{\left({\text{x}}_{\text{i}},{\text{y}}_{\text{i}}\right)\in {\text{S}}_{\text{xy}}}\omega \left({\text{x}}_{\text{i}},{\text{y}}_{\text{i}}\right)\frac{{\left(\text{I}\left({\text{x}}_{\text{i}},{\text{y}}_{\text{i}}\right)-\text{ L}\left(\text{x},\text{y}\right)\right)}^{2}}{\text{L}{\left(\text{x},\text{y}\right)}^{2}}}$$

Thirdly, the inhibitory weights *W*_Θ(x, y)_, W_L(x, y)_, W_C(x, y)_ were computed based on the center-surround mechanisms at each location for each feature, in which ${\overset{\bar{\Theta }}{\;}}_{\text{CRF}}$(x, y) was the orientation vector computed by Gaussian weighted averaging of Θ(x,y) in the region of CRF. The distance - related weighting function was denoted as W_d_, which meant that the strength of surround inhibition decreased with the increasing distance from the CRF center.

(6) $$\;{\text{W}}_{\;\Theta }\left(\text{x},\text{y}\right)=\exp\left(-\frac{{\left\|{{\;}^{\bar{\Theta }}}_{\text{CRF}}(\text{x},\text{y}){-}^{\bar{\Theta }}{\;}_{\text{NCRF}}(\text{x},\text{y})\right\|}^{2}}{2\sigma_{\Delta \theta }^{2}}\right)\;$$

(7) $${\text{W}}_{\text{L}}\left(\text{x},\text{y}\right)=\sum_{\left({\text{x}}_{\text{i}},{\text{y}}_{\text{i}}\right)}\exp\left(-\frac{\left|\text{L}\left(\text{x},\text{y}\right)-\text{L}({\text{x}}_{\text{i}},{\text{y}}_{\text{i}})\;\right|{\;}^{2}}{2\sigma_{\Delta l}^{2}}\right){\text{W}}_{\text{d}}\left({\text{x}}_{\text{i}}-\text{x},{\text{y}}_{\text{i}}-y\right)$$

(8) $${\text{W}}_{\text{C}}\left(\text{x},\text{y}\right)=\sum_{\left({\text{x}}_{\text{i}},{\text{y}}_{\text{i}}\right)}\exp\left(-\frac{{\left|\text{C}\left(\text{x},\text{y}\right)-\text{C}({\text{x}}_{\text{i}},{\text{y}}_{\text{i}})\;\right|}^{2}}{2\sigma_{\Delta \text{c}}^{2}}\right){\text{W}}_{\text{d}}\left({\text{x}}_{\text{i}}-\text{x},{\text{y}}_{\text{i}}-\text{y}\right)$$

Then, these three weights were integrated into a unified weight W_com_ based on a scale - guided combination strategy, where N(·) was a linear normalization operator.

(9) $$\begin{array}{l} {\text{W}}_{\text{com}}\left(\text{x},\text{y}\right)\\ =\{\begin{array}{l} \max\left({\text{W}}_{\;\Theta ,}{\text{W}}_{\text{L}},{\text{W}}_{\text{C}}\right)\left(\text{x},\text{y}\right),\text{ N}\left(\text{E}\left(\text{x},\text{y};\;\sigma \right)\right)-\text{N}\left(\text{E}\left(\text{x},\text{y};\;2\sigma \right)\right)>0\\ \min\left({\text{W}}_{\;\Theta ,}{\text{W}}_{\text{L}},{\text{W}}_{\text{C}}\right)\left(\text{x},\text{y}\right),\text{ N}\left(\text{E}\left(\text{x},\text{y};\;\sigma \right)\right)-\text{N}\left(\text{E}\left(\text{x},\text{y};\;2\sigma \right)\right)\leq 0 \end{array}\; \end{array}$$

Finally, the final response Res(x, y) was calculated based on the final inhibitory weight.

(10) $$\begin{array}{l} \text{Res}\left(\text{x},\text{y}\right)=\text{H}(\text{E}\left(\text{x},\text{y};\;\sigma \right)-\alpha \cdot {\text{W}}_{\text{com}}\left(\text{x},\text{y}\right)\cdot \text{E}\left(\text{x},\text{y};\;\sigma \right)\\ \;*\;{\text{W}}_{\text{d}}\left(\text{x},\text{y}\right)) \end{array}$$

(11) $$\text{where},\text{ H}\left(\text{z}\right)=\{\begin{array}{c} 0\text{ z}<0\\ \text{z z}\geq 0 \end{array}\;$$

**Figure 2:**
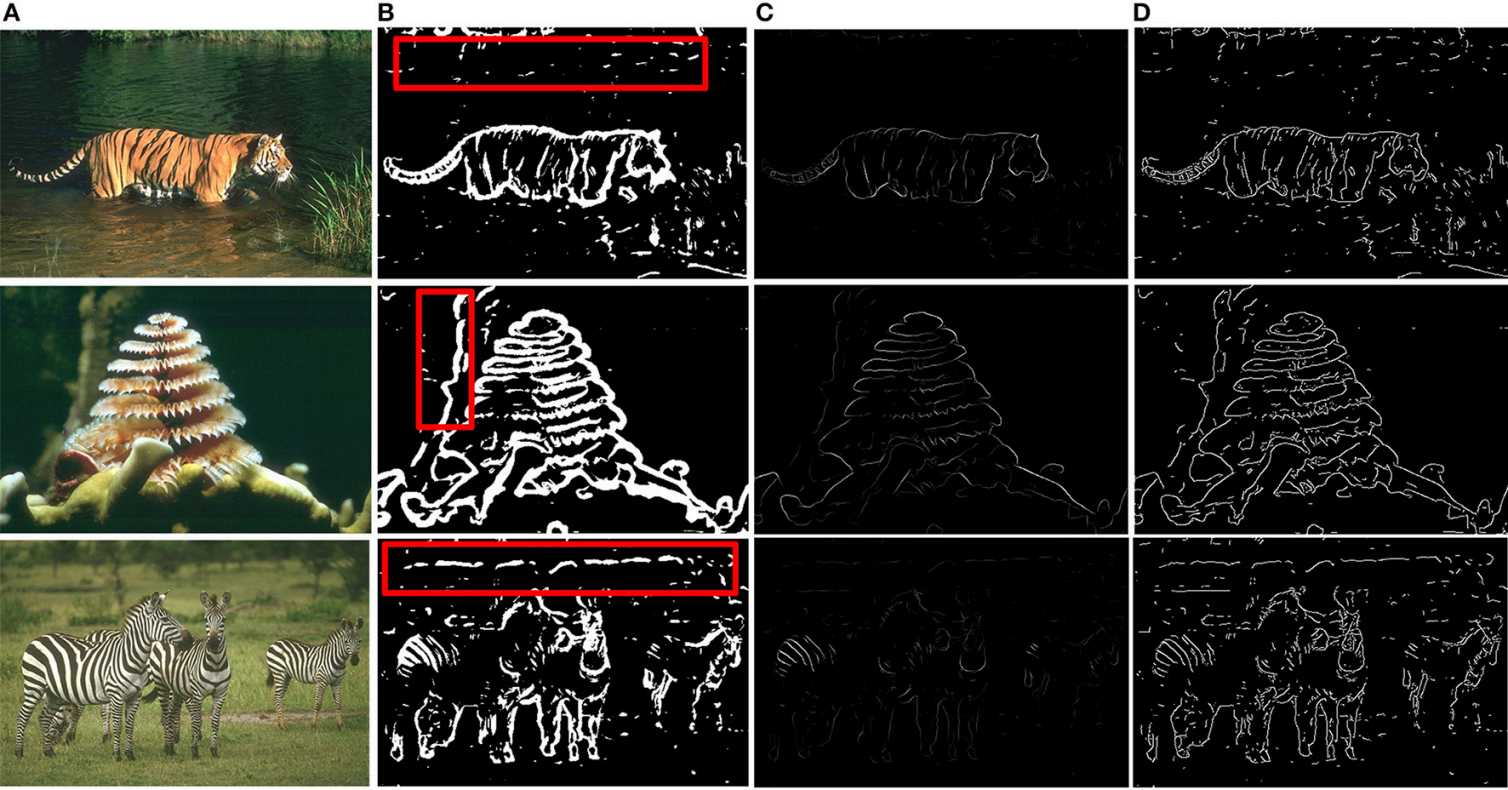
MCI results on natural images. **(A)** Input images. **(B)** Final contour responses without post-processing. The contour includes some unwanted textures located in red box. **(C)** Real-valued probability of contours after non-max suppression. **(D)** The binary images (containing values 0 or 1) after hysteresis thresholding.

Based on the above framework, MCI is applied to obtain the contours of natural images. Figure 2 gives some contour extracting results of MCI. Figure 2A represents input images, Figure 2B is final contour response without post-processing, Figure 2C represents the real-valued probability of contours after non-max suppression, Figure 2D is the binary image (containing values 0 or 1) after hysteresis thresholding. From the red box in Figure 2B, we can easily see that some texture contours obtained by the MCI do not belong to the real one. Table 1 shows the running time for every image in Figure 2, and the average time consuming of whole database (including 200 images for testing). And Table 1 also shows the runtime for every MCI step while the size of the input image is 481 × 321. It nearly takes 15 s to process an image, far from the processing speed of the human visual system. And the inhibitory weights at each location are computed for each feature, which consumes lots of time and does not compare to the fast and effective information processing in the human visual system. So, we propose an improved model, sMCI.

**Table 1:** The runtime of each step for MCI and the whole time on some images and the average time of BSDS 500.

	**Items**	**Runtime/s**
MCI steps	Inhibition Weights	14.93
	Extract Local Cues	0.78
	CRF Responses	0.25
	Weights Combination	0.23
	Salient Contour Extraction	0.05
Images	Image1	15.1
	Image2	15.4
	Image3	15.1
	BSDS500(200 test images)	15.5

### The sMCI model

#### Prior filtering and uniform sampling

To accelerate MCI, we improved it from two facts, which are prior filtering and uniform sampling. We will first introduce the process of prior filtering.

**Figure 3:**
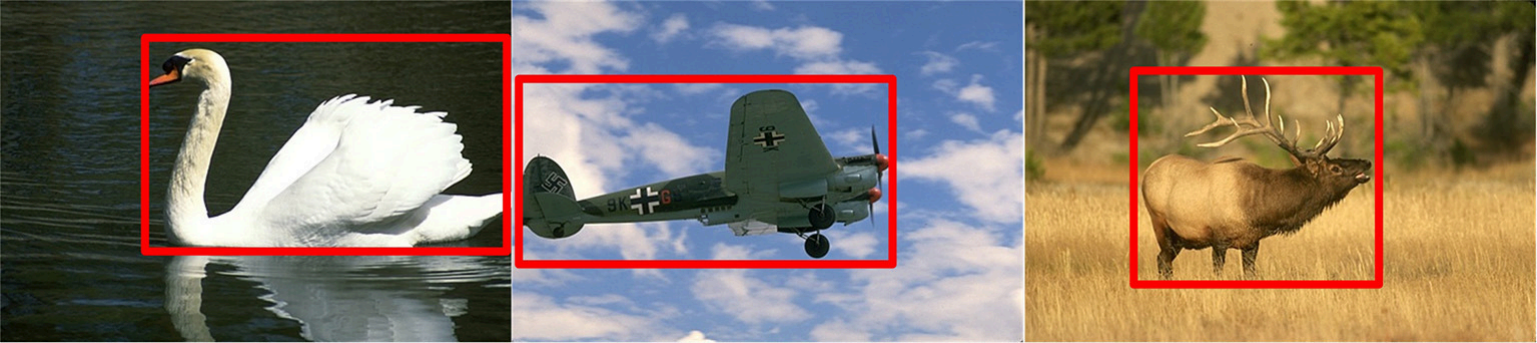
The mechanism of the prior filtering: most of the true contours are located in the red box, with low percentages in the whole image.

As shown in Figure 3, the salient object is located in the red box, which occupies a small portion relative to the whole image. Meanwhile, the contours of the object are salient relative to the background. Therefore, based on these observations, we only select the pixels, with their response value above 30% of the largest response after filtered with Gabor, to speed up the calculation of the inhibitory weights. The computing process is as the following:

(12) $${\text{W}}_{\Theta }\left(\text{x},\text{y}\right)=\{\begin{array}{ll} 1 & \text{ Loc}\left(\text{x},\text{y}\right)\;=0\\ \exp\left(-\frac{{\left\|{{\;}^{\bar{\Theta }}}_{\text{CRF}}(\text{x},\text{y}){-}^{\bar{\Theta }}{\;}_{\text{NCRF}}(\text{x},\text{y})\right\|}^{2}}{2\sigma_{\Delta \theta }^{2}}\right) & \text{ Loc}\left(\text{x},\text{y}\right)\;=1 \end{array}$$

where $\text{Loc}\left(\text{x},\text{y}\right)=\{\begin{array}{c} 0\text{ E}\left(\text{x},\text{y};\;\sigma \right)-30\%\;*\;{\text{E}}_{\max}<0\\ 1\text{ E}\left(\text{x},\text{y};\;\sigma \right)-30\%\;*\;{\text{E}}_{\max}\geq 0 \end{array}\;$, in which E_max_ represents the largest one of the entire image CRF responses.

Another observation is that the characteristics of adjacent neurons response have strong correlations which suggests that their responses are similar (Kohn, 2005). Based on this fact, there is no need to calculate the inhibitory weights of all neurons for each input image. The inhibitory responses of all neurons can be approximated by those of partial neurons.

**Figure 4:**
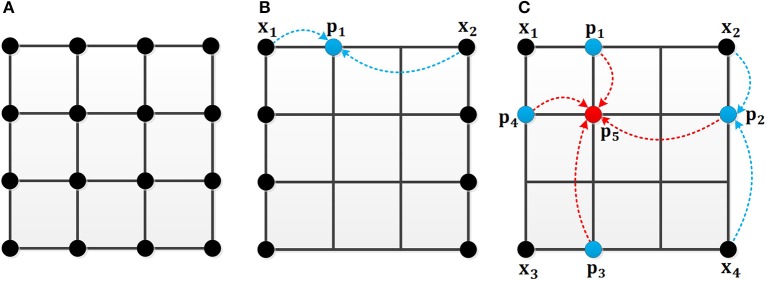
The mechanism of the uniform sampling. **(A)** The inhibitory weights at each location need to be calculated. **(B)** The uniform sampling in the x-direction. Only the weights at the black points need to be calculated. The blue points can be represented by the nearby black points. **(C)** The uniform sampling in x, y direction. The blue points can be calculated from the nearby black points. The red point can be obtained by the nearby blue points.

The other way to speed up the computation is sampling. This paper presents two sampling methods: sampling in one direction and in both directions. The detailed steps of two uniform sampling methods are as follows:

As shown in Figure 4, the black point in Figure 4A represents the location in the image. In the original MCI algorithm, the inhibitory weights are calculated at each location for every feature. For the uniform sampling in the x-direction, we just need to calculate the inhibitory weights of the black points in Figure 4B, and the inhibitory weights of the remaining points are obtained by the weighted sum of the nearby points. For example, the weight of the blue point can be obtained based on the two black points whose weights are known. Meanwhile, based on the biological mechanism that the influence of nearby neurons is greater than the one of distant neurons, the calculation formula of the inhibitory weights of the missing blue point in Figure 4B is in Equation (13):

(13) $$\;{\text{W}}_{{\text{p}}_{1}}=\frac{2}{3}\times {\text{W}}_{{\text{x}}_{1}}+\frac{1}{3}\times {\text{W}}_{{\text{x}}_{2}}$$

where W_p_1__ denotes the unknown weight of blue point, W_x_1__ and W_x_2__ represent the black points whose weights have been calculated. This also applies to the sampling in the y-direction.

In Figure 4C, an illustration is given to clarify the sampling process in both x and y directions. For a 4 × 4 image, only the weights of four black points are computed. The weights of the blue points are computed by two black points, and the weight of the red point can be represented by the weights of the four blue points. The calculation of inhibition weights of the missing blue points in Figure 4C is given in Equation (14):

(14) $${\text{W}}_{{\text{p}}_{2}}=\frac{2}{3}\times {\text{W}}_{{\text{x}}_{2}}+\frac{1}{3}\times {\text{W}}_{{\text{x}}_{4}}$$

and then the calculation of the weight of the missing red point is obtained by equation (15).

(15) $$\begin{array}{l} {\text{W}}_{{\text{p}}_{5}}=\frac{1}{3}\times {\text{W}}_{{\text{p}}_{1}}+\frac{1}{3}\times {\text{W}}_{{\text{p}}_{4}}+\frac{1}{6}\times {\text{W}}_{{\text{p}}_{2}}+\frac{1}{6}\times {\text{W}}_{{\text{p}}_{3}}\\ \;=\frac{4}{9}\times {\text{W}}_{{\text{x}}_{1}}+\frac{2}{9}\times {\text{W}}_{{\text{x}}_{2}}+\frac{2}{9}\times {\text{W}}_{{\text{x}}_{3}}+\frac{1}{9}\times {\text{W}}_{{\text{x}}_{4}} \end{array}$$

Where W_x_1__, W_x_2__, W_x_3__ and W_x_4__ represent the black points whose weights are known, W_p_1__, W_p_2__, W_p_3__, and W_p_4__denote the unknown weights of blue points, W_p_5__ denotes the unknown weight of the red point.

Finally, the prior filtering and uniform sampling are combined to further accelerate the speed of the method. To avoid losing too much real contour information, the following fusion method is adopted: for an image, we first select the pixels with their values above 10% of the largest response after filtered with Gabor, and then sample these pixels uniformly to further shorten the running time and ensure the integrity of the contour information.

#### Sparse coding

After accelerating the algorithm, we propose a method based on the biological mechanism to suppress the unwanted texture as shown in Figure 2.

Barlow (1981) has made a statistical and comprehensive analysis of the total number of cells in the visual pathway of macaques, which are shown in Table 2. The number of neurons in the lateral geniculate nucleus (LGN) is almost equal to the number of neurons in the ganglion, and the number of cells in the V1 region is much higher than that of the retina and the LGN. This comparison suggests that the responses of the V1 neurons have sparse properties. For the human visual system, sparse coding is crucial in encoding the input image, which can effectively suppress the redundant information. The local area containing some repeated textures will have a weak sparse response and the region including a stable boundary usually has a strong sparse response. Therefore, some unwanted contour noises can be effectively excluded based on the sparseness measure.

**Table 2:** Statistical data in the visual pathway of macaques (Unit: million) (Barlow, 1981).

**Ganglion**	**LGN**	**V1**
1.1	1.1–2.3	130–235

In this paper, we compute the sparseness measure as mentioned in Kai-Fu Yang et al. (2015) and Hoyer (2004) to distinguish the texture region and the non-texture region. The formula is as follows

(16) $$\text{sparseness}\left(\text{x},\;\text{y};\;\vec{\text{h}}\right)=\frac{1}{\sqrt{\text{n}}-1}\left(\sqrt{\text{n}}-\frac{{\left\|\vec{\text{h}}\left(\text{x},\;\text{y}\right)\right\|}_{1}}{{\left\|\vec{\text{h}}\left(\text{x},\;\text{y}\right)\right\|}_{2}}\right)$$

where $\vec{\text{h}}\left(\text{x},\text{y}\right)$ denotes the gradient's magnitude histogram of a local region centered at (x, y), n denotes the dimensionality of $\vec{\text{h}}\left(\text{x},\text{ y}\right)$, and $||\vec{\text{h}}\left(\text{x},\text{ y}\right)|{|}_{\text{p}}$ denotes the *l*_p_ of $\vec{\text{h}}\left(\text{x},\text{ y}\right)$, such as $\left||\vec{\text{h}}\left(\text{x},\text{ y}\right)|{|}_{1}=\sum |{\text{h}}_{\text{i}}\right|$, $||\vec{\text{h}}\left(\text{x},\text{ y}\right)|{|}_{2}=\sqrt{\sum |{\text{h}}_{\text{i}}{|}^{2}}$.

Then, the final neuron response FinalRes can be obtained by combining the original response Res and the sparseness, which is calculated as follows:

(17) $$\text{FinalRes}=\text{Res}\cdot \text{sparseness}\left(\text{x},\text{y};\vec{\text{h}}\right)$$

## Experiments and results

To evaluate the effectiveness of the proposed model, we test it on the BSDS500. The quantitative performance is compared with the original MCI model.

### Experiment settings

We test our model on the Berkeley Segmentation Data Set (BSDS500) (Martin et al., 2001). The BSDS500 is a dataset provided by the Berkeley computer vision group for image segmentation or contour detection, which includes 200 training, 200 testing, and 100 validation images. Boundaries in each image are labeled by several workers and are averaged to form the ground truth.

The performance is evaluated by the F-score (Martin et al., 2004), which denotes the similarity of the detected contours between human subjects and the algorithms. It is defined as F-score = 2PR / (P + R), where P represents the precision, R represents the recall.

Table 3 summarizes the meanings of the parameters involved in models, for example, the factor α in equation 10 denotes the connection strength between the CRF and the non-CRF. The parameter settings adopted in the MCI and sMCI model are the same.

**Table 3:** Parameter interpretations and settings (Yang et al., 2014).

**Parameter interpretations**	**MCI setting**	**sMCI setting**
α: Surround inhibition factor, or the texture attenuation factor	5	5
σ_Δθ_: Inhibition sensitivity of the feature difference of orientation	0.2	0.2
σ_Δ*l*_: Inhibition sensitivity of the feature difference of luminance	0.05	0.05
σ_Δ*c*_: Inhibition sensitivity of the feature difference of luminance contrast	0.05	0.05
*p*: Fraction of candidate edge pixels that should be retained in the contour edge map during hysteresis thresholding	1	1
*ws*: Size of window for sparseness measure	–	5

### Results of prior filtering and uniform sampling

**Figure 5:**
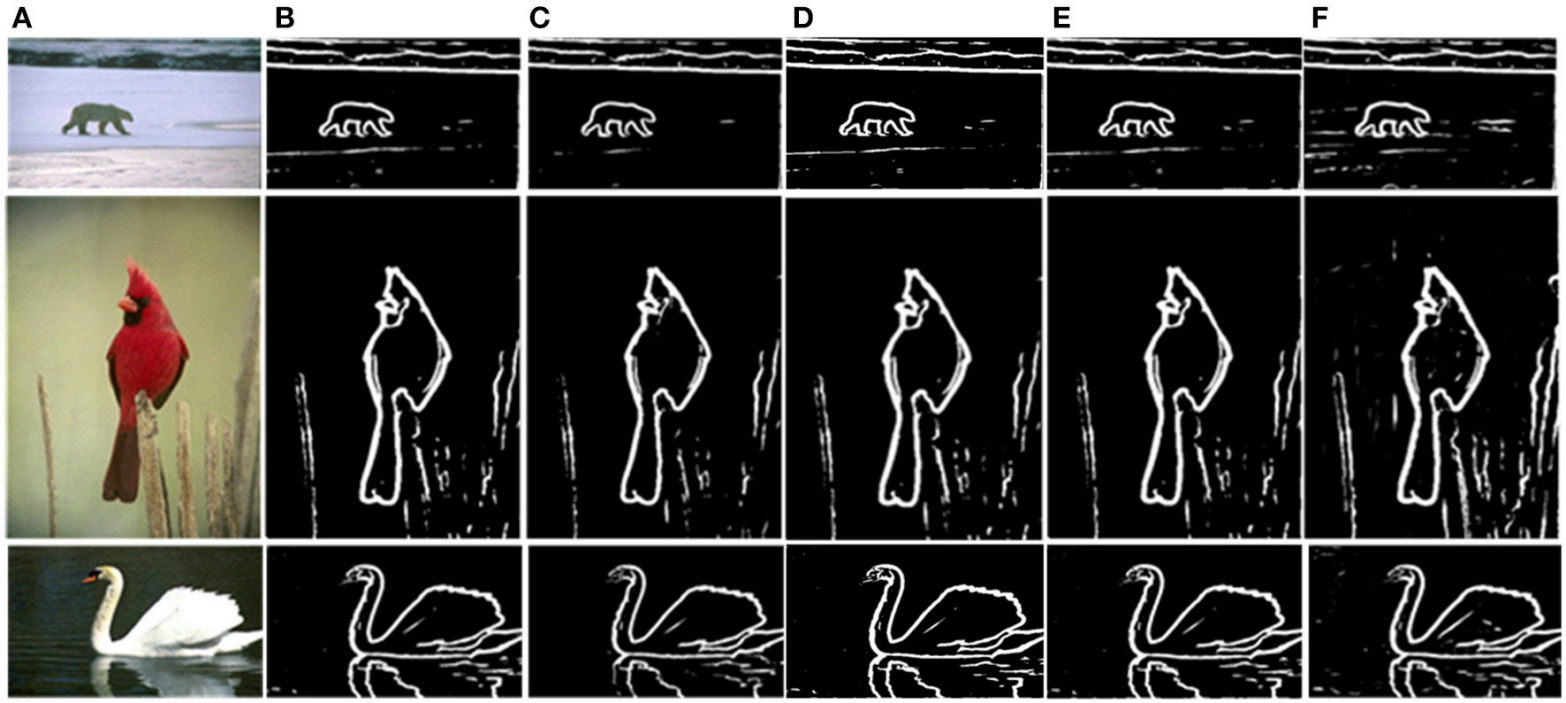
Comparison of experimental results. **(A)** Input images. **(B)** MCI results. **(C)** Prior filtering results. **(D)** Uniform sampling in the x-direction. **(E)** Uniform sampling in x, y direction. **(F)** Combined method.

**Figure 6:**
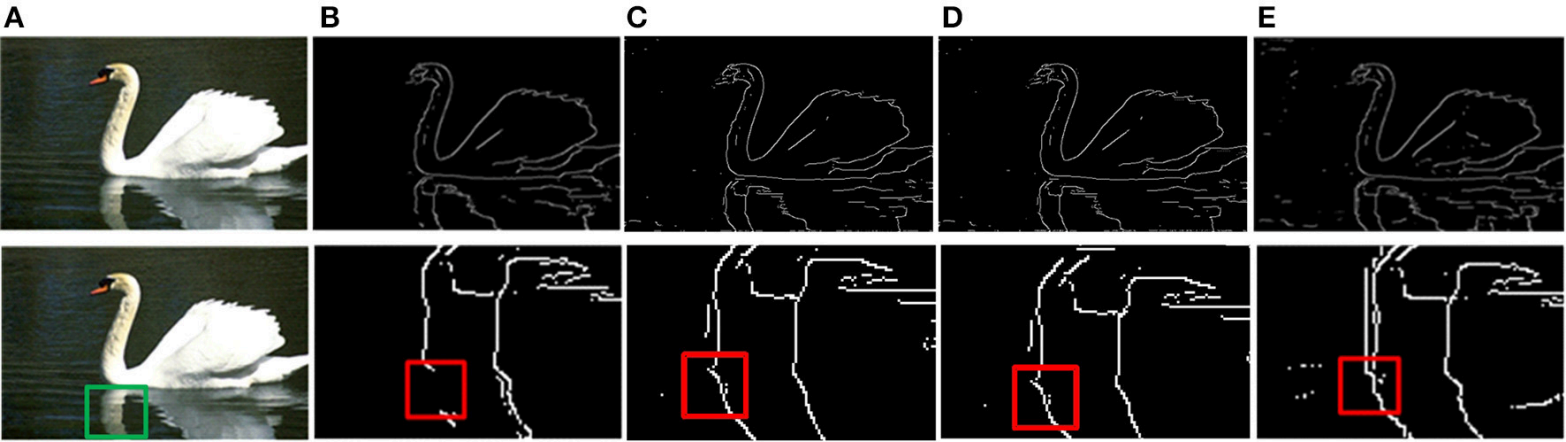
Results of three methods. **(A)** Input images. **(B)** Contour results after prior filtering with 30% largest responses. **(C)** Contour results after uniform sampling in the x-direction. **(D)** Contour results after uniform sampling in x, y direction. **(E)** Contour results after combined method.

The prior filtering adopts 30% of the largest responses as the threshold, whereas prior filtering in combined method adopts 10% of the largest responses. We compare the results of prior filtering, uniform sampling and the combined one with the original MCI algorithm and the results are shown in Figure 5. The F-score results and the running time are shown in Table 4. From these results, we can find that running time of the prior filtering method is relatively short but gets a lower F-score value. We amplify patch in the green box of Figure 6A and show it in Figures 6B–E. We can clearly see that some contours in red box extracted by prior filtering are lost depicted in Figure 6B. However, contours extracted by uniform sampling methods are complete, shown in Figures 6C,D. The running time is nearly the same if only sampling in one direction, and the same for the accuracy. However, the performance of uniform sampling in one direction outperforms sampling in both x and y directions, although the latter is superior to the former in running time. So, the combined method adopts sampling in one direction. And the result shows that the combined method can shorten the running time and keep the performance.

**Table 4:** Evaluation results and the runtime on BSDS 500 of the original MCI algorithm, the prior filtering, the uniform sampling in the x-direction, the uniform sampling in the y-direction, the uniform sampling in both directions, the combined method.

**Algorithms**	***F*-score**	**Running time/s**
MCI	0.627	3062
The prior filtering	0.617	1433
Uniform sampling in x direction	0.627	1530
Uniform sampling in y direction	0.626	1580
Uniform sampling in both directions	0.623	1319
The combined method	0.627	1208

### Results of sparse coding and final evaluation

**Figure 7:**
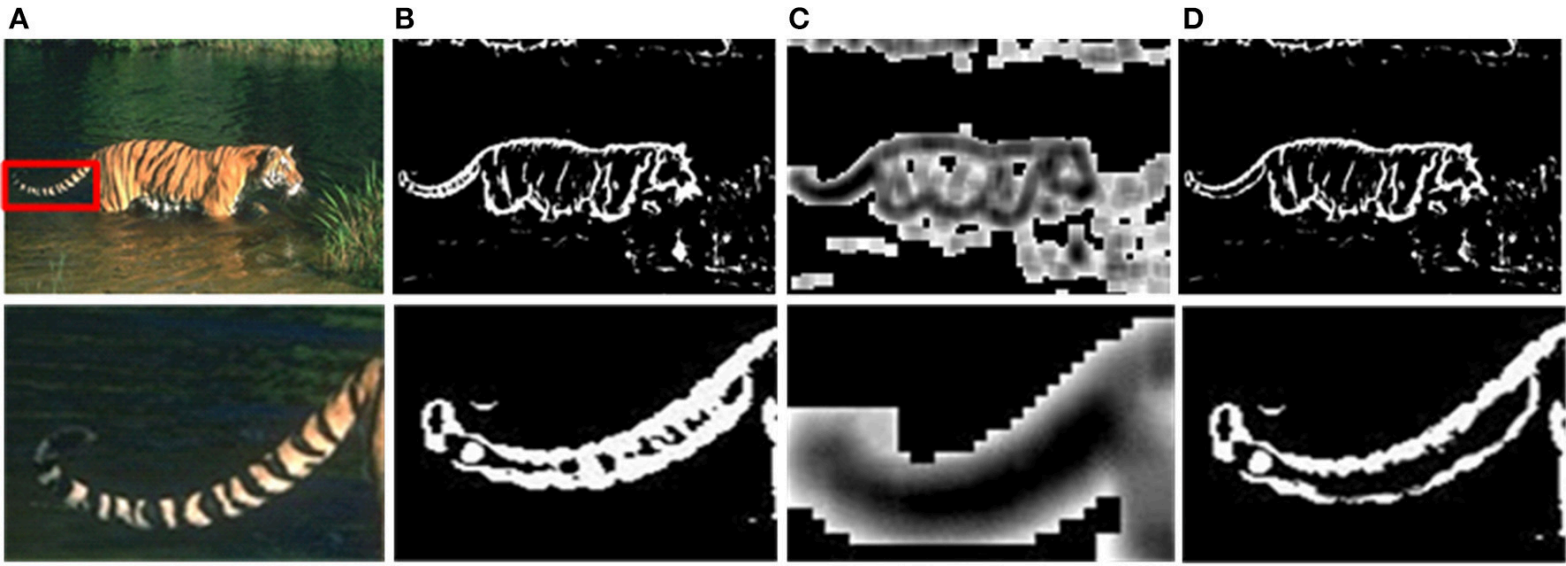
Results after sparse coding. **(A)** Input images. **(B)** Responses of the sMCI before sparse coding. **(C)** Sparseness responses. **(D)** Final responses after sparse coding.

Although the above method can solve the problem of time-consuming effectively, there are still unnecessary contour noises in sMCI results. Therefore, we use the sparse coding to suppress the unwanted edges. The experimental results of sparseness are shown in Figure 7, including the whole contour results and details. As shown in Figure 7B, the textures on tiger's tail are unwanted edges, and the sparse response is weak at that location illustrated in Figure 7C. By the process of sparse coding, the unwanted texture at the tail is suppressed shown in Figure 7D.

**Figure 8:**
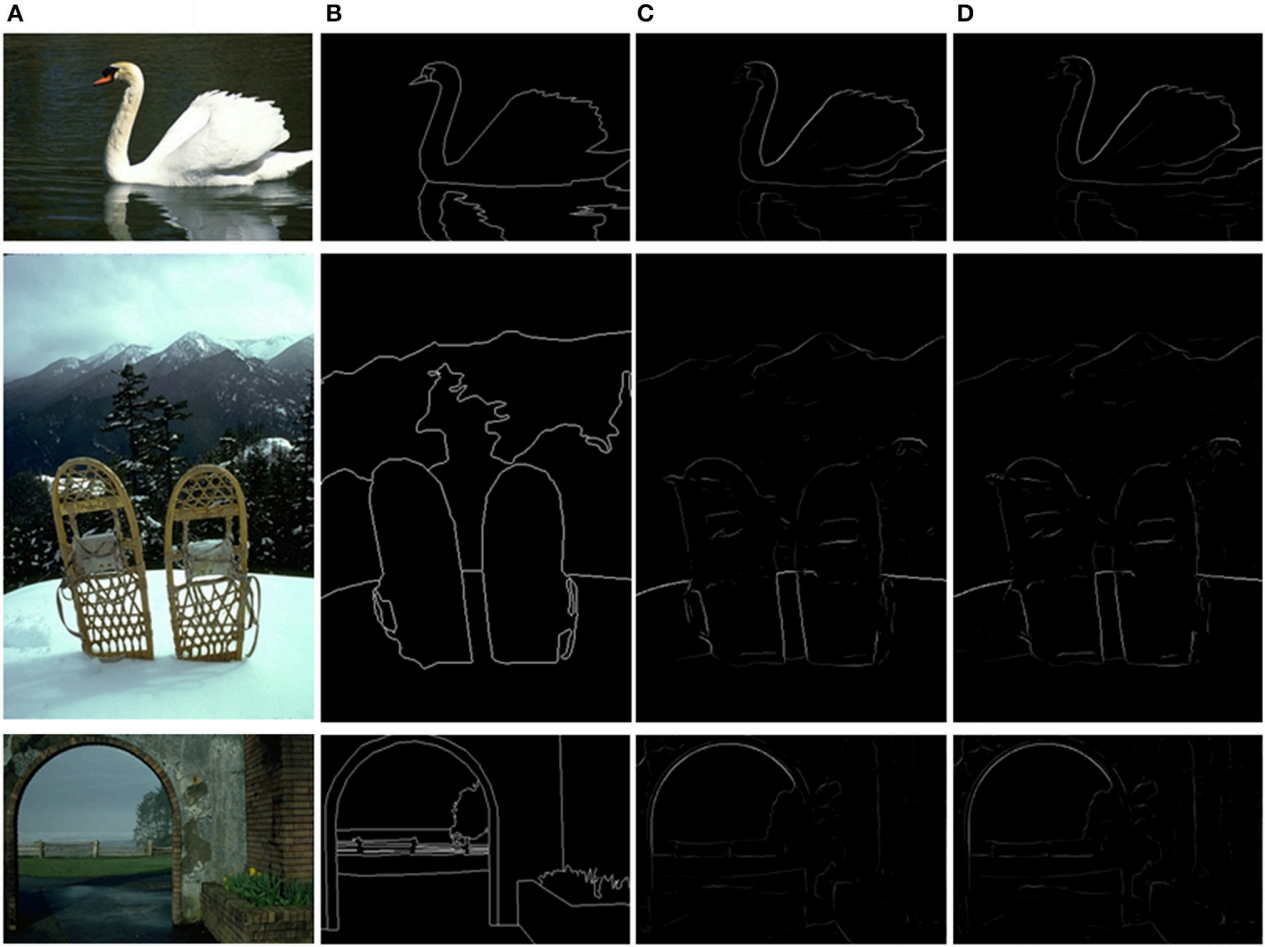
Evaluation images and results by MCI and sMCI. **(A)** Input images. **(B)** Ground truth. **(C)** MCI results (*F*-score = 0.627). **(D)** sMCI results (*F*-score = 0.629).

The final results after non-maxima suppression (Canny, 1986) between MCI and sMCI models are shown in Figure 8. Figure 8A is the original image, Figure 8B represents the ground truth, Figure 8C is the MCI result and Figure 8D is the sMCI result. The *F*-score values are shown in Table 5.

**Table 5:** Evaluation results for MCI and sMCI after sparse coding.

**Algorithms**	***F*-score**	**Running time/s**
MCI	0.627	3062
sMCI	0.629	1470

The experimental results demonstrate that sMCI model effectively reduces the running time by 52% without degrading the performance in contour accuracy.

## Discussion

Based on the MCI algorithm, we proposed a fast contour detection model, inspired by the information processing mechanism in the human primary vision system. The prior filtering and uniform sampling effectively reduced the running time. And the sparse coding served to exclude the unwanted textures. The results on BSDS500 showed the competitive performance and fastness of the model.

The bright spots of our work can be summarized below. (a) We adopt the prior filtering based on the knowledge of human behavioral psychology, which can focus on the area containing the desired contours. (b) Uniform sampling is introduced based on the biological mechanism that nearby neurons often have highly correlated responses and thus include redundant information. We only calculate the weights of the partial feature rather than the whole images and reconstructs the whole feature responses based on properties between nearby neurons. (c) Sparse coding is introduced in the model, which provided an effective way to suppress the unwanted edges. The experimental results showed that the method can decrease the running time as well as keeping the accuracy of the contour detection.

However, the mechanism of the algorithm still has a gap with the human visual system. Therefore, how to optimize the model based on more biological mechanisms is our next step.

From the bottom-up mechanism, we can integrate more underlying features. In our work, we only consider features such as the orientation, the luminance, and the luminance contrast. However, the color contrast is also a crucial feature for contour detection. And in the human visual system, the color information is modulated by color-opponent mechanisms. One important extension of our current model is how to utilize the cue of color in an effective way. In future, we can design a framework combining the center-surround and color-opponent mechanisms to optimize the performance of contour detection.

From the top-down mechanism, we can integrate the feedback mechanism which plays an important modulatory role to the V1 neurons' responses. In fact, it is very challenging to extract the salient object boundaries in complex environments. And a feedback process can provide attentional support to salient or behaviorally-relevant features.

In summary, the model we proposed based on the biological mechanisms in this paper can both keep the accuracy and decrease the time-consuming. In the study, we can find that the neuroscience research promotes the development of the model research. In the future, the current research will be extended with more neuroscience results. From these studies, we also hope to understand the inner mechanisms of the information processing of the human brain.

## Author contributions

XK, QK, and YZ designed the work. XK and QK contributed to the experiments. XK, QK, YZ, and BX contributed to the results analysis. XK, QK, YZ, and BX contributed to the writing of the manuscript. The version of work is approved to be published by BX, YZ, QK, and XK.

### Conflict of interest statement

The authors declare that the research was conducted in the absence of any commercial or financial relationships that could be construed as a potential conflict of interest.
